# Concordance of HER2 status between core needle biopsy and surgical resection specimens of breast cancer: an analysis focusing on the HER2-low status

**DOI:** 10.1007/s12282-024-01585-3

**Published:** 2024-04-21

**Authors:** Sei Na, Milim Kim, Yujun Park, Hyun Jung Kwon, Hee-Chul Shin, Eun-Kyu Kim, Mijung Jang, Sun Mi Kim, So Yeon Park

**Affiliations:** 1 Department of Pathology, Seoul National University Bundang Hospital, Seoul National University College of Medicine, 82, Gumi-ro 173 Beon-gil, Bundang-gu, Seongnam, Gyeonggi 13620 Republic of Korea; 2 Department of Surgery, Seoul National University Bundang Hospital, Seoul National University College of Medicine, Seongnam, Gyeonggi Republic of Korea; 3 Department of Radiology, Seoul National University Bundang Hospital, Seoul National University College of Medicine, Seongnam, Gyeonggi Republic of Korea

**Keywords:** Breast cancer, HER2-low, Core needle biopsy, Concordance

## Abstract

**Background:**

Human epidermal growth factor receptor 2 (HER2)-low status has recently gained attention because of the potential therapeutic benefits of antibody–drug conjugates (ADCs) in breast cancer patients. We aimed to investigate the concordance of HER2 status between core needle biopsy (CNB) and subsequent surgical resection specimens focusing on the HER2-low status.

**Methods:**

This retrospective study was conducted in 1,387 patients with invasive breast cancer whose HER2 status was evaluated in both CNB and surgical resection specimens. The discordance rates between CNB and surgical resection specimens and the clinicopathological features associated with HER2 status discordance were analyzed.

**Results:**

The overall concordance rates of HER2 status between CNB and surgical resection specimens were 99.0% (*κ* = 0.925) for two-group classification (negative vs. positive) and 78.5% (*κ* = 0.587) for three-group classification (zero vs. low vs. positive). The largest discordance occurred in CNB-HER2-zero cases with 42.8% of them reclassified as HER2-low in surgical resection. HER2 discordance was associated with lower histologic grade, tumor multiplicity, and luminal A subtype. In multivariate analysis, tumor multiplicity and estrogen receptor (ER) positivity were independent predictive factors for HER2-zero to low conversion.

**Conclusions:**

Incorporation of HER2-low category in HER2 status interpretation reduces the concordance rate between CNB and surgical resection specimens. Tumor multiplicity and ER positivity are predictive factors for conversion from HER2-zero to HER2-low status. Therefore, HER2 status should be re-evaluated in resection specimens when considering ADCs in tumors exhibiting multiplicity and ER positivity.

## Introduction

Breast cancer is a heterogeneous disease, and accurate determination of basic biomarkers including estrogen receptor (ER), progesterone receptor (PR), and human epidermal growth factor receptor 2 (HER2) is crucial for selecting appropriate treatment for breast cancer patients. Immunohistochemistry (IHC) and in situ hybridization (ISH) are standard methods for evaluating HER2 status in breast cancer [1]. HER2-targeted therapy is exclusively effective in HER2-positive (IHC 3 + and/or ISH-positive) breast cancers which account for 15–20% of invasive breast cancers [2, 3]. Thus, approximately 80–85% of invasive breast cancers are classified as HER2-negative (IHC 0, IHC 1 + , and IHC 2 + /ISH-negative) and are not eligible for HER2-targeted therapy.

Traditionally, HER2-targeted agents were not applicable for breast cancers with intermediate HER2 expression levels often referred to as HER2-low (IHC 1 + or IHC 2 + /ISH-negative) [4]. However, recent observations with novel anti-HER2 compounds suggest that a subset of HER2-low breast cancers may benefit from HER2-targeted therapies. For example, the DESTINY-Breast04 trial, an open-label phase III study, showed a significant improvement in survival outcomes among advanced/metastatic HER2-low breast cancer patients treated with an antibody–drug conjugate (ADC), trastuzumab deruxtecan, compared with those treated with the chemotherapy regimen selected by physicians [5]. Subsequently, the American Society of Clinical Oncology (ASCO)/College of American Pathologists (CAP) revised their guidelines for HER2 testing. They incorporated footnotes specifically addressing HER2-low tumors to guide identification and treatment of trastuzumab deruxtecan-eligible patients [6].

Core needle biopsy (CNB) is less invasive and more cost-effective than excisional biopsy, and it is currently recommended as the first-line diagnostic modality [7]. Many studies have explored the reliability of biomarker testing using CNB compared with ground-truth surgical biomarker results and have reported favorable outcomes [8–10]. Although evaluation of HER2 status using CNB was considered reliable, such results were based on a binary classification system, which distinguishes HER2-positive from HER2-negative tumors [11–13]. The results may differ when HER2 status is classified as HER2-positive, HER2-low, or HER2-zero. Lu et al. observed a decrease in HER2 concordance rate between CNB and surgical resection specimens when incorporating HER2-low, with *κ* value of 0.684 [14]. Chen et al. also reported moderate concordance of HER2 status between CNB and surgical resection specimens with three-tiered classification including HER2-low (*κ* = 0.57) [15]. As the current ASCO/CAP guidelines use a 10% cutoff point for HER2 IHC scoring [16], CNB may not represent the HER2 status of the tumor if only a small portion of the tumor shows HER2 staining. Moreover, HER2 staining patterns are not homogeneous in some cases [17, 18]. Recent studies have reported the limited diagnostic value of CNB in identifying HER2-low breast cancers [14, 15].

This study aimed to investigate the concordance of HER2 status between CNB and subsequent surgical resection specimens with a specific focus on the HER2-low status to validate diagnostic accuracy of CNB for determining HER2 status. We also aimed to identify the factors associated with discordance to guide the HER2 re-evaluation in surgical resection specimens.

## Materials and methods

### Patient selection

Data of patients diagnosed with invasive breast cancer who underwent surgery at Seoul National University Bundang Hospital between January 1, 2021, and December 31, 2022, were collected retrospectively. Patients who were histologically diagnosed with invasive breast cancer using CNB and subsequent surgical resection and whose both preoperative CNB and surgical resection specimens were available to standard biomarker testing were eligible for the study. Patients treated with primary systemic therapy (PST) such as neoadjuvant chemotherapy, targeted therapy, or endocrine therapy were excluded.

**Fig. 1:**
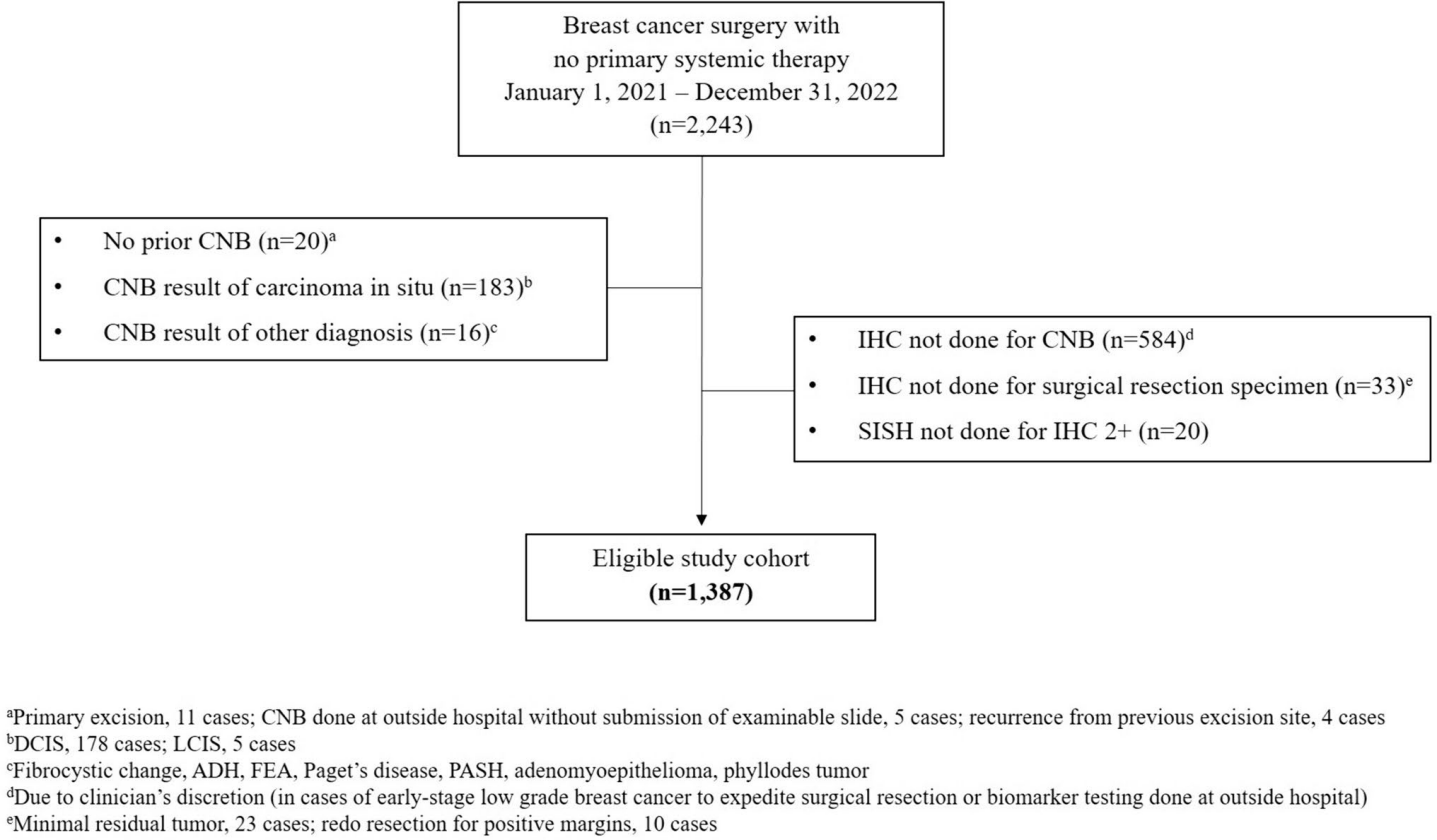
Inclusion criteria. *CNB* core needle biopsy, *IHC* immunohistochemistry, *SISH* silver in situ hybridization, *DCIS* ductal carcinoma in situ, *LCIS* lobular carcinoma in situ, *ADH* atypical ductal hyperplasia, *FEA* flat epithelial atypia, *PASH* pseudoangiomatous stromal hyperplasia

During this period, 2,243 patients with invasive breast cancer underwent surgery without PST. Of them, 856 patients were excluded due to the following reasons: preoperative histologic confirmation was not performed in 20 patients, CNB results indicated in situ carcinoma or other entities in 199 patients, standard biomarker status was not evaluated preoperatively in 584 patients due to clinician’s discretion (in cases of early-stage low-grade breast cancer to expedite surgical resection or biomarker testing done in another institution), the biomarker status was not evaluated in surgical resection specimens in 33 patients, and HER2 ISH was not performed in 20 patients with equivocal (2 +) HER2 IHC result (Fig. 1). Consequently, 1,387 patients were included in the analysis.

### Clinicopathological information

Clinical and pathological data were retrieved from the electronic medical records and pathological reports during the study. All CNB and surgical resection specimens were initially diagnosed by experienced breast pathologists. For biopsy specimens, data on the number of tissue cores, histologic subtype (based on the World Health Organization classification criteria), histologic grade (based on the Bloom and Richardson grading system), and the presence or absence of in situ carcinoma were collected. For surgical resection specimens, comprehensive information pertaining to the histological diagnosis, including histologic subtype, tumor size, T stage, N stage, histologic grade, lymphovascular invasion, tumor-infiltrating lymphocytes, and tumor multiplicity, were documented and used for this study. For multifocal or multicentric breast cancer, data of the largest index tumor was used for analyses. Preoperative CNB and surgical resection specimens were matched based on radiologic findings such as size and location. The clinicopathological characteristics of the patients included in this study are summarized in Table 1.

**Table 1:** Clinicopathological characteristics of tumors according to HER2 status

Clinicopathological characteristics	Total (*n* = 1,387)	HER2 status			*p* value			
		Zero (*n* = 354)	Low (*n* = 929)	Positive (*n* = 104)	Three groups	HER2-zero vs. HER2-low*	HER2-low vs. HER2-positive*	HER2-zero vs. HER2-positive*
Age at diagnosis								
< 50 years old	510 (36.8)	115 (32.5)	362 (39.0)	33 (31.7)	0.053	0.096	0.450	1.000
≥ 50 years old	877 (63.2)	239 (67.5)	567 (61.0)	71 (68.3)				
Histologic subtype								
Invasive carcinoma of NST	1192 (85.9)	297 (83.9)	797 (85.8)	98 (94.2)	0.050	1.000	0.102	0.063
Invasive lobular carcinoma	117 (8.4)	31 (8.9)	84 (9.0)	2 (1.9)				
Others	78 (5.6)	26 (7.3)	48 (5.2)	4 (3.8)				
T stage								
T1	899 (64.8)	230 (65.0)	603 (64.9)	66 (63.5)	0.956	1.000	1.000	1.000
T2–4	488 (35.2)	124 (35.0)	326 (35.1)	38 (36.5)				
N stage								
N0	919 (66.3)	246 (69.5)	608 (65.4)	65 (62.5)	0.024	0.117	0.117	0.939
N1–3	398 (28.7)	86 (24.3)	283 (30.5)	29 (27.9)				
Not evaluated	70 (5.0)	22 (6.2)	38 (4.1)	10 (9.6)				
Histologic grade								
Low to intermediate	1006 (72.5)	244 (68.9)	731 (78.7)	31 (29.8)	< 0.001	< 0.001	< 0.001	< 0.001
High	381 (27.5)	110 (31.1)	198 (21.3)	73 (70.2)				
Lymphovascular invasion								
Present	503 (36.3)	128 (36.2)	334 (36.0)	41 (39.4)	0.783	1.000	1.000	1.000
Absent	884 (63.7)	226 (63.8)	595 (64.0)	63 (60.6)				
Tumor multiplicity								
Present	416 (30.0)	98 (27.7)	289 (31.1)	29 (27.9)	0.434	0.696	1.000	1.000
Absent	971 (70.0)	256 (72.3)	640 (68.9)	75 (72.1)				
Tumor-infiltrating lymphocytes								
< 10%	1,056 (76.1)	272 (76.8)	730 (78.6)	54 (51.9)	< 0.001	1.000	< 0.001	< 0.001
10–50%	280 (20.2)	71 (20.0)	174 (18.7)	35 (33.7)				
≥ 50%	51 (3.7)	11 (3.2)	25 (2.7)	15 (14.4)				
Molecular subtype								
Luminal A	752 (54.2)	185 (52.3)	567 (61.0)	0 (0.0)	< 0.001	< 0.001	< 0.001	< 0.001
Luminal B	486 (35.0)	113 (31.9)	308 (33.2)	65 (62.5)				
HER2 +	39 (2.8)	0 (0.0)	0 (0.0)	39 (37.5)				
Triple negative	110 (7.9)	56 (15.8)	54 (5.8)	0 (0.0)				
Estrogen receptor								
Positive	1,238 (89.3)	298 (84.2)	875 (94.2)	65 (62.5)	< 0.001	< 0.001	< 0.001	< 0.001
Negative	149 (10.7)	56 (15.8)	54 (5.8)	39 (37.5)				
Progesterone receptor								
Positive	1,107 (79.8)	270 (76.3)	784 (84.4)	53 (51.0)	< 0.001	0.003	< 0.001	< 0.001
Negative	280 (20.2)	84 (23.7)	145 (15.6)	51 (49.0)				
Ki-67 proliferation index								
< 20%	973 (70.2)	237 (66.9)	706 (76.0)	30 (28.8)	< 0.001	0.003	< 0.001	< 0.001
≥ 20%	414 (29.8)	117 (33.1)	223 (24.0)	74 (71.2)				

Numbers in parentheses indicate column percentage

*P* values were calculated by Chi-square test

^*^Corrections for multiple testing were performed with Bonferroni method, and adjusted *p* values are presented

### Immunohistochemical data

Immunohistochemical staining was performed in both CNB and surgical resection specimens to identify standard biomarkers, including ER, PR, HER2, and Ki-67. IHC staining procedures were performed on BenchMark XT autostainer (Ventana Medical Systems, Tucson, AZ, USA) using ultraView Universal DAB Detection Kit (Ventana Medical Systems) with the following antibodies: ER (ready-to-use; clone SP1; Ventana Medical Systems), PR (ready-to-use; clone 1E2; Ventana Medical Systems), HER2 (ready-to-use; clone 4B5; Ventana Medical Systems), and Ki-67 (1:200; clone MIB-1; Dako, Carpinteria, CA, USA).

HER2 IHC status was determined according to the 2018 ASCO/CAP guideline: 0, no staining observed or incomplete membrane staining of weak intensity within ≤ 10% of tumor cells; 1 + , incomplete membrane staining of weak intensity in > 10% of tumor cells; 2 + , weak to moderate, complete membranous staining in > 10% of the tumor cells; and 3 + , complete and intense circumferential membrane staining in > 10% of tumor cells. For HER2 equivocal (2 +) cases, HER2 silver ISH (SISH) was performed using INFORM HER2 DNA and chromosome 17 probes (Ventana Medical Systems). HER2/CEP17 ratio ≥ 2.0 and average HER2 copy number ≥ 4.0 per cell was defined as HER2 SISH-positive. HER2/CEP17 ratio of < 2.0 and an average HER2 copy number of < 4.0 per cell was defined as HER2 SISH-negative. In other circumstances, HER2 IHC was reviewed with SISH, and the final HER2 status was assigned in accordance with the 2018 ASCO/CAP guidelines [16]. There has been no change in staining protocol for HER2 IHC using 4B5 clone in our country, that was recently reported in Japan [19].

Finally, HER2 status was classified as negative (IHC 0, 1 + , or 2 + /SISH-negative) or positive (IHC 2 + /SISH-positive or IHC 3 +) using a two-group classification system. Alternatively, HER2 status was classified as zero (IHC 0), low (IHC 1 + or IHC 2 + /SISH-negative), or positive (IHC 2 + /SISH-positive or IHC 3 +) using a three-group classification system. In addition, HER2 status was classified as IHC 0, IHC 1 + , IHC 2 + /SISH-negative, and IHC 2 + /SISH-positive or IHC 3 + using a four-group classification system. HER2 IHC results at the time of diagnosis were used for initial data collection. However, for cases with HER2 discordance between CNB and surgical resection, an experienced breast pathologist (SYP) reviewed the HER2 IHC slides to eliminate inter-observer variability.

ER and PR were considered positive if more than 1% of tumor nuclei showed staining [20]. For Ki-67 proliferation index, tumors with 20% or more positive tumor cells were regarded as having a high proliferative index.

Breast cancer subtypes were determined using standard biomarker profiles according to the 2013 St. Gallen International Expert Consensus [21]. Each subtype was defined as follows: luminal A (ER + , PR + , HER2 − , Ki-67 < 20%), luminal B (ER + , HER2 − and at least one of: Ki-67 ≥ 20% or PR < 20%; ER + , HER2 + , any Ki-67, any PR), HER2 + (ER − , PR − , HER2 +), and triple negative (ER − , PR − , HER2 −).

### Statistical analysis

SPSS version 26.0.0 for Windows (IBM Corporation, New York, USA) was used for statistical analyses. Pearson’s Chi-square test was used to compare the clinicopathological characteristics according to HER2 status. Corrections for multiple testing were performed using the Bonferroni method, and the adjusted *p* values were calculated. The concordance rates between the HER2 statuses of CNB and surgical resection specimens were analyzed using kappa statistics. Pearson’s Chi-square test was used to compare the clinicopathological parameters associated with discordance in HER2 status between CNB and surgical resection specimens. The differences in the number of CNB cores and tumor sizes were analyzed using independent sample *t*-tests. Logistic regression analysis using a backward stepwise selection method was used to analyze the factors associated with the conversion of HER2-zero to HER2-low status in the resected specimens. The odds ratios (OR) and 95% confidence intervals (CIs) were calculated for significant variables. All *p* values were two-sided, and *p* values less than 0.05 were considered statistically significant.

## Results

### Clinicopathological characteristics according to HER2 status

HER2 IHC results for surgical resection specimens were as follows: score of 0 in 354 cases, 1 + in 501 cases, 2 + in 467 cases, and 3 + in 65 cases. Among the 467 cases with an IHC score of 2 + , 428 were HER2 SISH-negative, and 39 were HER2 SISH-positive. Of the 1,387 cases, 354 (25.5%) were HER2-zero, 929 (67.0%) were HER2-low, and 104 (7.5%) were HER2-positive.

We first compared the clinicopathological characteristics of the tumors according to HER2 status (Table 1). Overall, significant differences were observed in N stage (*p* = 0.024), histologic grade (*p* < 0.001), level of tumor-infiltrating lymphocytes (*p* < 0.001), molecular subtype (*p* < 0.001), ER status (*p* < 0.001), PR status (*p* < 0.001), and Ki-67 proliferation index (*p* < 0.001) according to HER2 status.

When the HER2-low and HER2-zero groups were compared, low histological grade (*p* < 0.001), ER positivity (*p* < 0.001), PR positivity (*p* = 0.003), and low Ki-67 proliferation index (*p* = 0.003) were more frequently observed in the HER2-low group than in the HER2-zero group. The HER2-low group showed lower histologic grade (*p* < 0.001), fewer tumor-infiltrating lymphocytes (*p* < 0.001), higher ER positivity rate (*p* < 0.001), higher PR positivity rate (*p* < 0.001), and lower Ki-67 proliferation index (*p* < 0.001) than the HER2-positive group.

### CNB-surgical resection concordance of HER2 status

Using a binary classification of the HER2-negative and HER2-positive groups, the overall concordance rate was 99.0% (1,373/1,387) with a kappa coefficient of 0.925 (*p* < 0.001; Table 2). Of the 1291 cases with HER2-negative results on CNB, 11 cases were HER2-positive in subsequent surgical resection specimens. Of these, three cases showed HER2 heterogeneity in the surgically resected specimen. Among 96 cases which were HER2-positive on CNB, three were eventually classified as HER2-negative in the surgical resection specimen, and all of them were reported to have low levels of HER2 amplification in the CNB specimens.

**Table 2:** Concordance of HER2 status in CNB and surgical resection specimen

HER2 status (two groups)		Surgical resection		*κ*-coefficient	*p*-value
		Negative (*n* = 1283)	Positive (*n* = 104)		
CNB	Negative (*n* = 1291)	1280 (99.1)	11 (0.9)	0.925	< 0.001
	Positive (*n* = 96)	3 (3.1)	93 (96.9)		

HER2 status (three groups)		Surgical resection			*κ*-coefficient	*p*-value
		Zero (*n* = 354)	Low (*n* = 929)	Positive (*n* = 104)		
CNB	Zero (*n* = 498)	283 (56.8)	213 (42.8)	2 (0.4)	0.587	< 0.001
	Low (n = 793)	71 (9.0)	713 (89.9)	9 (1.1)		
	Positive (*n* = 96)	0 (0.0)	3 (3.1)	93 (96.9)		

HER2 status (four groups)		Surgical resection				*κ*-coefficient	*p*-value
		IHC 0 (*n* = 354)	IHC 1 + (*n* = 501)	IHC 2 + /ISH– (*n* = 428)	IHC 2 + /ISH + & IHC 3 + (*n* = 104)		
CNB	IHC 0 (*n* = 498)	283 (56.8)	172 (34.5)	41 (8.2)	2 (0.4)	0.443	< 0.001
	IHC 1 + (*n* = 542)	69 (12.7)	274 (50.6)	193 (35.6)	6 (1.1)		
	IHC 2 + /ISH- (*n* = 251)	2 (0.8)	55 (21.9)	191 (76.1)	3 (1.2)		
	IHC 2 + /ISH + and IHC 3 + (*n* = 96)	0 (0.0)	0 (0.0)	3 (3.1)	93 (96.9)		

Numbers in parentheses indicate row percentage

*IHC* immunohistochemistry, *ISH* in situ hybridization

**Fig. 2:**
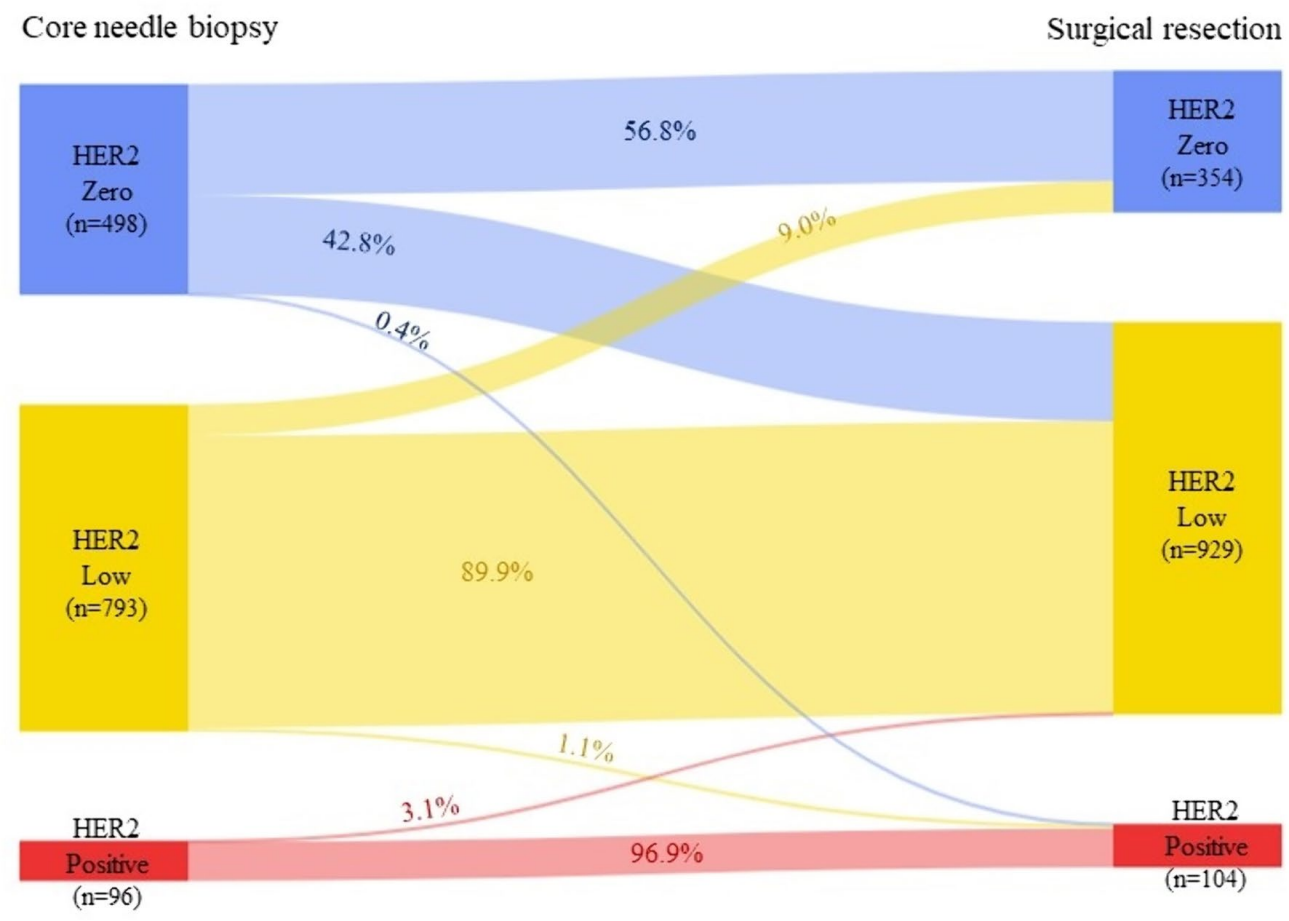
Sankey diagram showing HER2 status concordance between core needle biopsy and surgical resection specimen

Based on the three-group classification system (HER2-zero, low, and positive), the overall concordance rate was 78.5% (1,089/1,387) with a kappa coefficient of 0.587 (*p* < 0.001), indicating moderate consistency (Table 2; Fig. 2). The greatest discordance was reported in CNB-HER2-zero cases of which 42.8% (213/498) were later revealed to have a HER2-low status in the surgical resection specimens. The second-largest discordance was observed in CNB-HER2-low cases where 9.0% (71/793) was found to be HER2-zero in the surgical resection specimens.

When using the four-group classification system based on the HER2 IHC scores and SISH results (IHC 0, IHC 1 + , IHC 2 + /SISH-negative, IHC 2 + /SISH-positive or IHC 3 +), the overall concordance rate further decreased to 60.6% (841/1,387) with a kappa coefficient of 0.443 (*p* < 0.001) (Table 2). The largest discordance was observed in CNB-HER2 IHC 1 + cases where 35.6% (193/542) was later revealed to be HER2 IHC 2 + /SISH-negative in the surgical resection specimens. The second-largest discordance was observed in CNB-HER2 IHC 0 cases where 34.5% (172/498) was later identified as having HER2 IHC 1 + in the surgical resection specimens.

### Parameters associated with HER2 discordance

**Fig. 3:**
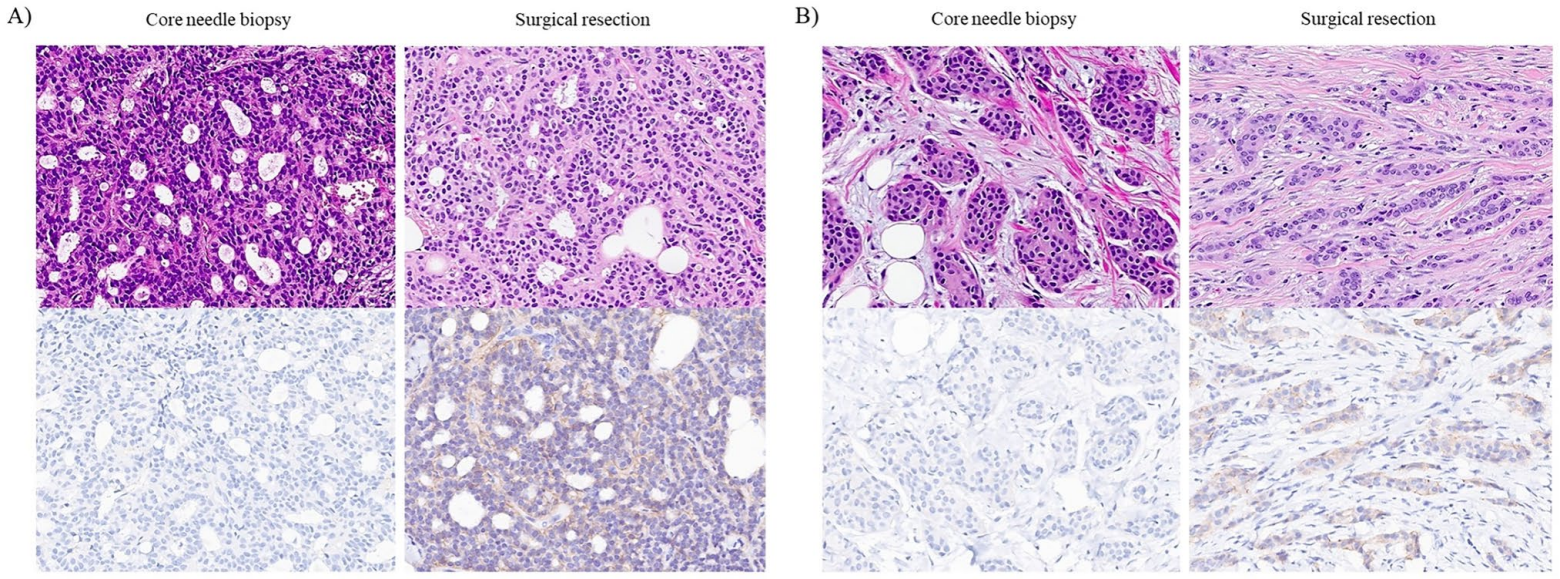
Histologic and immunohistochemical findings of core needle biopsy (CNB) HER2-zero to surgical resection HER2-low converted cases. **A** A low-grade invasive breast cancer with HER2 immunohistochemistry (IHC) score 0 in CNB reveals HER2 IHC score 1 + in surgical resection specimen. **B** An intermediate grade invasive breast cancer with HER2 IHC score 0 in CNB reveals HER2 IHC score 1 + in surgical resection specimen

According to the three-group classification system, 298 cases (21.5%) showed discordance in HER2 status between CNB and subsequent surgical resection specimens (Fig. 3). We analyzed the clinicopathological parameters associated with discordance in HER2 status between CNB and surgical resection specimens (Table 3). Compared to the concordant cases, the discordant cases had a lower histologic grade (*p* = 0.014), more multiplicity (*p* < 0.001), and higher rate of the luminal A subtype (*p* = 0.035). Hormone receptor status and Ki-67 proliferation index did not reveal a significant association.

**Table 3:** Clinicopatholgical parameters associated with discordance of HER2 status between CNB and surgical resection specimen using three-group classification system

Clinicopatholgical parameters	Concordant (n = 1,089)	Discordant (n = 298)	*p*-value
Age at diagnosis			
< 50 years old	390 (35.8)	120 (40.3)	0.158
≥ 50 years old	699 (64.2)	178 (59.7)	
Number of biopsy cores			
Mean ± SD	4.16 ± 1.32	4.19 ± 1.55	0.716
Histologic subtype			
Invasive carcinoma of no special type	941 (86.4)	251 (84.2)	0.576
Invasive lobular carcinoma	90 (8.3)	27 (9.1)	
Others	58 (5.3)	20 (6.7)	
Size of tumor (cm)			
Mean ± SD	2.02 ± 1.23	1.99 ± 1.09	0.833
T stage			
T1	710 (65.2)	189 (63.4)	0.570
T2–4	379 (34.8)	109 (36.6)	
N stage			
N0	721 (66.2)	198 (66.4)	0.824
N1–3	311 (28.6)	87 (29.2)	
Not evaluated	57 (5.2)	13 (4.4)	
Histologic grade			
Grade I–II (low to intermediate)	773 (71.0)	233 (78.2)	0.014
Grade III (high)	316 (29.0)	65 (21.8)	
Lymphovascular invasion			
Present	392 (36.0)	111 (37.2)	0.690
Absent	697 (64.0)	187 (62.8)	
Tumor multiplicity			
Present	302 (27.7)	114 (38.3)	< 0.001
Absent	787 (72.3)	184 (61.7)	
Tumor-infiltration lymphocytes			
< 10%	820 (75.3)	236 (79.2)	0.162
≥ 10%	269 (24.7)	62 (20.8)	
Molecular subtype			
Luminal A	585 (53.7)	167 (56.0)	0.035
Luminal B	381 (35.0)	105 (35.2)	
HER2 +	38 (3.5)	1 (0.3)	
Triple negative	85 (7.8)	25 (8.4)	
Estrogen receptor			
Positive	966 (88.7)	272 (91.3)	0.204
Negative	123 (11.3)	26 (8.7)	
Progesterone receptor			
Positive	866 (79.5)	241 (80.9)	0.607
Negative	223 (20.5)	57 (19.1)	
Ki-67 proliferation index			
< 20%	756 (69.4)	217 (72.8)	0.256
≥ 20%	333 (30.6)	81 (27.2)	

Numbers in parentheses indicate column percentage

*P* values were calculated by Chi-square test or independent sample t-test

Considering the tendency of CNB-HER2-zero tumors to be reclassified as HER2-low on surgical resection specimens, the clinicopathological parameters associated with HER2-zero to low conversion were determined (Table 4). Tumors with CNB-HER2-zero to surgical resection-HER2-low conversion showed younger age at diagnosis, lower histologic grade, and higher rate of tumor multiplicity compared to HER2-zero concordant tumors (*p* = 0.017, *p* = 0.001 and *p* = 0.001, respectively). ER and PR positivity rates were higher (*p* = 0.001 and *p* = 0.048, respectively), while Ki-67 proliferation index was lower (*p* = 0.024) in the tumors with HER2-zero to low conversion.

**Table 4:** clinicopathological parameters associated with CNB-HER2-zero to surgical resection-HER2-low converted cases in comparison with HER2-zero concordant cases

clinicopathological parameters	Zero to zero (*n* = 283)	Zero to low (*n* = 213)	*p*-value
Age at diagnosis			
< 50 years old	99 (35.0)	97 (45.5)	0.017
≥ 50 years old	184 (65.0)	116 (54.5)	
Number of biopsy cores			
Mean ± SD	4.24 ± 1.36	4.18 ± 1.54	0.655
Histologic subtype			
Invasive carcinoma of no special type	234 (82.7)	174 (81.7)	0.927
Invasive lobular carcinoma	25 (8.8)	21 (9.9)	
Others	24 (8.5)	18 (8.5)	
Size of tumor (cm)			
Mean ± SD	2.05 ± 1.20	2.06 ± 1.11	0.935
T stage			
T1	176 (62.2)	127 (59.6)	0.562
T2–4	107 (37.8)	86 (40.4)	
N stage			
N0	194 (68.6)	140 (65.7)	0.253
N1–3	70 (24.7)	64 (30.0)	
Not evaluated	19 (6.7)	9 (4.2)	
Histologic grade			
Grade I–II (low to intermediate)	191 (67.5)	171 (80.3)	0.001
Grade III (high)	92 (32.5)	42 (19.7)	
Lymphovascular invasion			
Present	106 (37.5)	84 (39.4)	0.653
Absent	177 (62.5)	129 (60.6)	
Tumor multiplicity			
Present	79 (27.9)	89 (41.8)	0.001
Absent	204 (72.1)	124 (58.2)	
Tumor-infiltrating lymphocytes			
< 10%	216 (76.3)	172 (80.8)	0.237
≥ 10%	67 (23.7)	41 (19.2)	
Molecular subtype			
Luminal A	151 (53.4)	132 (62.0)	0.004
Luminal B	86 (30.4)	67 (31.5)	
Triple negative	46 (16.3)	14 (6.6)	
Estrogen receptor			
Positive	237 (83.7)	199 (93.4)	0.001
Negative	46 (16.3)	14 (6.6)	
Progesterone receptor			
Positive	219 (77.4)	180 (84.5)	0.048
Negative	64 (22.6)	33 (15.5)	
Ki-67 proliferation index			
< 20%	186 (65.7)	160 (75.1)	0.024
≥ 20%	97 (34.3)	53 (24.9)	

Numbers in parentheses indicate column percentage

*P* values were calculated by Chi-square test or independent sample *t*-test

Using univariate binary logistic regression analysis, younger age (*p* = 0.018), low histological grade (*p* = 0.002), tumor multiplicity (*p* = 0.001), ER positivity (*p* = 0.001), PR positivity (*p* = 0.049), and low Ki-67 proliferation index (*p* = 0.025) were confirmed to be predictors of HER2-zero to low conversion in surgical resection specimens (Table 5**)**. In the multivariate analysis, tumor multiplicity (OR: 1.768, CI 1.209–2.586, *p* = 0.003) and ER positivity (OR: 2.580, CI 1.371–4.854, *p* = 0.003) were proven to be independent predictive factors for HER2-zero to low conversion (Table 5).

**Table 5:** Univariate and multivariate logistic regression analyses for factors associated with CNB-HER2-zero to surgical resection-HER2-low conversion

Clinicopathological parameters	Univariate analysis		Multivariate analysis	
	OR [95% CI]	*p*-value	OR [95% CI]	*p-*value
Age at diagnosis				
< 50 vs. ≥ 50 years old	0.643 [0.447–0.926]	0.018		
Histologic grade				
I and II vs. III	0.510 [0.335–0.776]	0.002		
Tumor-infiltrating lymphocyte				
< 10% vs. ≥ 10%	0.768 [0.496–1.190]	0.238		
Tumor multiplicity				
Absent vs. present	1.853 [1.272–2.700]	0.001	1.768 [1.209–2.586]	0.003
Estrogen receptor				
Negative vs. positive	2.759 [1.473–5.166]	0.002	2.580 [1.371–4.854]	0.003
Progesterone receptor				
Negative vs. positive	1.594 [1.002–2.535]	0.049		
Ki-67 proliferation index				
< 20% vs. ≥ 20%	0.635 [0.428–0.944]	0.025		

## Discussion

This study investigated concordance of HER2 status in 1,387 breast cancer patients with matched CNB-surgical resection specimens. The overall HER2 concordance rate between CNB and surgical resection specimens was excellent when using the conventional two-group classification (99.0%, κ = 0.925) but decreased to 78.5% (κ = 0.587) when using the three-group classification (HER2-zero, HER2-low, and HER2-positive). The discrepancy was largest for CNB-HER2-zero cases, in which 42.8% were reclassified as having HER2-low status, largely HER2 IHC 1 + , upon subsequent surgical resection.

Conversion of a significant proportion of tumors initially classified as CNB-HER2-zero into HER2-low status on surgical resection can be attributed to the use of the current semi-quantitative HER2 IHC scoring system and heterogeneity of HER2 expression. First, the distinction between HER2 IHC 0 and HER2 IHC 1 + may be arbitrary and vulnerable to inter-observer variation [18]. Although Karakas et al. reported a 70% concordance rate between HER2 IHC 0 and 1 + readings [22], Lambein et al. showed that 76% of the local HER2 IHC 0 cases were classified as HER2 IHC 1 + upon central reassessment [23]. Criteria adjustment and training, especially in distinguishing between HER2-0 and HER2 IHC 1 + , are essential for increasing the concordance of HER2-low results. Second, since heterogeneous HER2 expression is common in breast cancer [18], if the number of cores in a CNB is insufficient or the tumor is large, the CNB results may not represent the entire tumor. However, our data showed that the number of cores in biopsy specimens and tumor size did not differ between concordant and discordant cases; therefore, the degree of CNB as representative of the entire tumor may have less impact on discordance.

In our study, ER positivity was a characteristic of HER2-low breast cancers and was an independent indicator of HER2-zero to low conversion in the resected specimen. Previous studies have consistently reported that HER2-low breast cancer is characterized by higher incidence of hormone receptor positivity and luminal subtype [24–26]. Thus, our finding that ER positivity is a predictive factor for HER2-low status in resection specimens in CNB-HER2-zero cases may indicate that ER positivity is highly associated with HER2-low status. Otherwise, whether ER positivity is associated with increased regional heterogeneity of HER2 expression needs further investigations. In the current study, besides ER/PR positivity, low histologic grade and low Ki-67 proliferation index were associated with HER2-low breast cancers. Denkert et al. also reported that HER2-low breast cancers tended to show higher hormone receptor expression levels, lower cell proliferation, and lower tumor grade compared to HER2-zero breast cancers in a pooled analysis of 2,310 patients [24].

In this study, we showed that tumor multiplicity was related to HER2 discordance and was an independent predictor of HER2-zero to low conversion in resected specimens. Multicentric tumors (those within different quadrants) and multifocal tumors (those within the same quadrant) are regarded as “multiple breast cancer” if separated by normal or benign breast tissue [27]. For these cases, we matched the CNB with the resection specimen by correlating the preoperative radiologic findings such as size and location of tumors with the gross surgical findings. However, we cannot guarantee absolute accuracy of these matches. In addition, some studies reported discrepancies in biomarker status within individual tumors of multiple breast cancer, with discordance in HER2 status observed in 5%–16% of cases [28–30]. Previously, we observed an inter-lesional heterogeneity in the standard biomarker expression and genomic alterations in a significant proportion of multiple breast cancers despite the histological features of multiple tumors being identical [31]. Thus, biomarker testing from one index tumor may not be sufficient to determine the characteristics of a patient’s disease and eligibility for targeted therapy.

In our cohort, 70.0% of the patients had HER2-low breast cancer, which was higher than the previously reported 45–55% of breast cancer cases [4]. This difference could be attributable to the current treatment standards where patients with HER2-positive breast cancer most likely undergo PST; thus, a portion of them was inevitably excluded in this study. In addition, the fact that even many patients without PST were excluded in our study should be taken into account before drawing any conclusions.

The present study evaluated 1,387 breast cancer patients to elucidate concordance in the HER2 status between CNB and surgical resection specimens, with a focus on the HER2-low status. However, this study has some limitations. First, although HER2 testing and interpretation were performed in accordance with the 2018 updated ASCO/CAP guidelines and the diagnostic breast pathologists who performed the procedure were sufficiently trained, pre-analytic factors such as tissue fixation and cold ischemic time which may affect HER2 IHC status were not considered. Second, although we meticulously matched CNB and surgical resection specimens and exclusively utilized data from the index tumors, it is conceivable that different tumors were inadvertently paired.

In summary, our study confirmed that incorporation of the HER2-low category into HER2 status interpretation in breast cancer resulted in decreased concordance between CNB and surgical resection specimens. Notably, our findings revealed tumor multiplicity and ER positivity as pivotal predictive factors for conversion of HER2-zero status in CNB into HER2-low status in subsequent surgical resection. In clinical practice, our study underscores the need of re-evaluating HER2 status in surgical resection specimens in instances of tumor multiplicity or ER positivity, especially when considering ADC treatment options. Such process is critical in ensuring precise HER2 classification which would in turn, lead to optimal treatment for breast cancer patients.

## Data Availability

The datasets used and/or analyzed during the current study are available from the corresponding author on reasonable request.

## References

[1] Ahn S, Woo JW, Lee K, Park SY (2020). HER2 status in breast cancer: changes in guidelines and complicating factors for interpretation. J Pathol Transl Med.

[2] Slamon DJ, Clark GM, Wong SG, Levin WJ, Ullrich A, McGuire WL (1987). Human breast cancer: correlation of relapse and survival with amplification of the HER-2/neu oncogene. Science.

[3] Tandon AK, Clark GM, Chamness GC, Ullrich A, McGuire WL (1989). HER-2/neu oncogene protein and prognosis in breast cancer. J Clin Oncol.

[4] Tarantino P, Hamilton E, Tolaney SM, Cortes J, Morganti S, Ferraro E (2020). HER2-low breast cancer: pathological and clinical landscape. J Clin Oncol.

[5] Modi S, Jacot W, Yamashita T, Sohn J, Vidal M, Tokunaga E (2022). Trastuzumab deruxtecan in previously treated HER2-low advanced breast cancer. N Engl J Med.

[6] Wolff AC, Somerfield MR, Dowsett M, Hammond EH, Hayes DF, McShane LM (2023). Human epidermal growth factor receptor 2 testing in breast cancer American Society of Clinical Oncology-College of American Pathologists Guideline Update. Arch Pathol Lab Med..

[7] Bruening W, Fontanarosa J, Tipton K, Treadwell JR, Launders J, Schoelles K (2010). Systematic review: comparative effectiveness of core-needle and open surgical biopsy to diagnose breast lesions. Ann Intern Med..

[8] Shanmugalingam A, Hitos K, Hegde S, Al-Mashat A, Pathmanathan N, Edirimmane S (2022). Concordance between core needle biopsy and surgical excision for breast cancer tumor grade and biomarkers. Breast Cancer Res Treat.

[9] Burge CN, Chang HR, Apple SK (2006). Do the histologic features and results of breast cancer biomarker studies differ between core biopsy and surgical excision specimens?. Breast.

[10] Park SY, Kim KS, Lee TG, Park SS, Kim SM, Han W (2009). The accuracy of preoperative core biopsy in determining histologic grade, hormone receptors, and human epidermal growth factor receptor 2 status in invasive breast cancer. Am J Surg.

[11] Lee AH, Key HP, Bell JA, Hodi Z, Ellis IO (2012). Concordance of HER2 status assessed on needle core biopsy and surgical specimens of invasive carcinoma of the breast. Histopathology.

[12] Lorgis V, Algros MP, Villanueva C, Chaigneau L, Thierry-Vuillemin A, Nguyen T (2011). Discordance in early breast cancer for tumour grade, estrogen receptor, progesteron receptors and human epidermal receptor-2 status between core needle biopsy and surgical excisional primary tumour. Breast.

[13] Jeong YS, Kang J, Lee J, Yoo T-K, Kim SH, Lee A (2020). Analysis of the molecular subtypes of preoperative core needle biopsy and surgical specimens in invasive breast cancer. J Pathol Transl Med.

[14] Lu Y, Zhu S, Tong Y, Fei X, Jiang W, Shen K (2022). HER2-low status is not accurate in breast cancer core needle biopsy samples: an analysis of 5610 consecutive patients. Cancers (Basel)..

[15] Chen R, Qi Y, Huang Y, Liu W, Yang R, Zhao X (2023). Diagnostic value of core needle biopsy for determining HER2 status in breast cancer, especially in the HER2-low population. Breast Cancer Res Treat.

[16] Wolff AC, Hammond MEH, Allison KH, Harvey BE, Mangu PB, Bartlett JMS (2018). Human epidermal growth factor receptor 2 testing in breast cancer: American Society of Clinical Oncology/College of American Pathologists Clinical Practice Guideline Focused Update. J Clin Oncol.

[17] Seol H, Lee HJ, Choi Y, Lee HE, Kim YJ, Kim JH (2012). Intratumoral heterogeneity of HER2 gene amplification in breast cancer: its clinicopathological significance. Mod Pathol.

[18] Zhang H, Katerji H, Turner BM, Audeh W, Hicks DG (2022). HER2-low breast cancers: incidence, HER2 staining patterns, clinicopathologic features, MammaPrint and BluePrint genomic profiles. Mod Pathol.

[19] Horimoto Y, Onagi H, Watanabe J, Hayashi T (2024). HER2 staining of breast cancer is reduced under the current protocol of Ventana PATHWAY anti-HER-2/neu (4B5). Pathol Int.

[20] Allison KH, Hammond MEH, Dowsett M, McKernin SE, Carey LA, Fitzgibbons PL (2020). Estrogen and progesterone receptor testing in breast cancer: ASCO/CAP guideline update. J Clin Oncol.

[21] Goldhirsch A, Winer EP, Coates AS, Gelber RD, Piccart-Gebhart M, Thürlimann B (2013). Personalizing the treatment of women with early breast cancer: highlights of the St Gallen International Expert Consensus on the Primary Therapy of Early Breast Cancer 2013. Ann Oncol.

[22] Karakas C, Tyburski H, Turner BM, Wang X, Schiffhauer LM, Katerji H (2023). Interobserver and interantibody reproducibility of her2 immunohistochemical scoring in an enriched HER2-low-expressing breast cancer cohort. Am J Clin Pathol.

[23] Lambein K, Van Bockstal M, Vandemaele L, Geenen S, Rottiers I, Nuyts A (2013). Distinguishing score 0 from score 1+ in HER2 immunohistochemistry-negative breast cancer: clinical and pathobiological relevance. Am J Clin Pathol.

[24] Denkert C, Seither F, Schneeweiss A, Link T, Blohmer JU, Just M (2021). Clinical and molecular characteristics of HER2-low-positive breast cancer: pooled analysis of individual patient data from four prospective, neoadjuvant clinical trials. Lancet Oncol.

[25] Schettini F, Chic N, Brasó-Maristany F, Paré L, Pascual T, Conte B (2021). Clinical, pathological, and PAM50 gene expression features of HER2-low breast cancer. npj Breast Cancer..

[26] Tarantino P, Jin Q, Tayob N, Jeselsohn RM, Schnitt SJ, Vincuilla J (2022). Prognostic and biologic significance of ERBB2-lowexpression in early-stage breast cancer. JAMA Oncol.

[27] Salgado R, Aftimos P, Sotiriou C, Desmedt C (2015). Evolving paradigms in multifocal breast cancer. Semin Cancer Biol.

[28] Choi Y, Kim EJ, Seol H, Lee HE, Jang MJ, Kim SM (2012). The hormone receptor, human epidermal growth factor receptor 2, and molecular subtype status of individual tumor foci in multifocal/multicentric invasive ductal carcinoma of breast. Hum Pathol.

[29] Chou S, Khan T, Mahajan H, Pathmanathan N (2015). Predicting discordant HER2 results in ipsilateral synchronous invasive breast carcinomas: experience from a single institution. Pathology.

[30] Boros M, Ilyes A, Nechifor Boila A, Moldovan C, Eniu A, Stolnicu S (2014). Morphologic and molecular subtype status of individual tumor foci in multiple breast carcinoma. A study of 155 cases with analysis of 463 tumor foci. Hum Pathol.

[31] Ahn S, Kim HJ, Kang E, Kim EK, Kim SH, Kim JH (2020). Genomic profiling of multiple breast cancer reveals inter-lesional heterogeneity. Br J Cancer.

